# Supplementary material for: Detailed molecular and epigenetic characterization of the pig IPEC-J2 and chicken SL-29 cell lines
Source: iScience. 2023 Feb 20;26(3):106252. doi: 10.1016/j.isci.2023.106252 (PMC10018572; doi:10.1016/j.isci.2023.106252)
Supplement: Data S1. Complete homer output for identified motifs in Pig IPECJ-2, related to Table 2 — Homer motif analysis results for histone modifications H3K4me1, H3K4me3, H3K27ac, and enhancer elements of pig IPECJ2 cell line. P-values >1e-10 are possible false positives. Within each folder (e.g. peak_files_CTCF) are the html files showing the identified motifs when using homer (e.g. homerResults.html). [file mmc2.zip › S5/Pig_IPECJ_2/peak_files_H3K4me1/knownResults.html]

peak\_files\_H3K4me1 - Homer Known Motif Enrichment Results


# Homer Known Motif Enrichment Results (peak\_files\_H3K4me1)

Homer *de novo* Motif Results  
Gene Ontology Enrichment Results  
Known Motif Enrichment Results (txt file)  
Total Target Sequences = 100389, Total Background Sequences = 100489

|  |  |  |  |  |  |  |  |  |  |  |  |
| --- | --- | --- | --- | --- | --- | --- | --- | --- | --- | --- | --- |
| Rank | Motif | Name | P-value | log P-pvalue | q-value (Benjamini) | # Target Sequences with Motif | % of Targets Sequences with Motif | # Background Sequences with Motif | % of Background Sequences with Motif | Motif File | SVG |
| 1 | A C T G C A T G T C G A A C G T A C T G C G T A A T C G A C G T G T A C C G T A G A C T A G T C | Fos(bZIP)/TSC-Fos-ChIP-Seq(GSE110950)/Homer | 1e-267 | -6.149e+02 | 0.0000 | 10591.0 | 10.55% | 7516.6 | 7.48% | motif file (matrix) | svg |
| 2 | C A T G C T A G T C G A A C G T A C T G C G T A T A G C C G A T T G A C C G T A A G C T G A T C | Fra2(bZIP)/Striatum-Fra2-ChIP-Seq(GSE43429)/Homer | 1e-259 | -5.984e+02 | 0.0000 | 8691.0 | 8.66% | 5949.6 | 5.92% | motif file (matrix) | svg |
| 3 | C T A G T C G A A C G T A C T G C G T A T A G C C G A T G T A C C G T A A G C T G A T C G T A C | Jun-AP1(bZIP)/K562-cJun-ChIP-Seq(GSE31477)/Homer | 1e-254 | -5.855e+02 | 0.0000 | 4357.0 | 4.34% | 2501.8 | 2.49% | motif file (matrix) | svg |
| 4 | A C T G C T A G T C G A C G A T C A T G G C T A A T C G C G A T G T A C G C T A A G C T G T A C | Fra1(bZIP)/BT549-Fra1-ChIP-Seq(GSE46166)/Homer | 1e-248 | -5.725e+02 | 0.0000 | 10133.0 | 10.09% | 7222.9 | 7.19% | motif file (matrix) | svg |
| 5 | C T A G T C G A C G A T A C T G C G T A T A C G A G C T T G A C G C T A A C G T G A T C T A G C | Fosl2(bZIP)/3T3L1-Fosl2-ChIP-Seq(GSE56872)/Homer | 1e-248 | -5.725e+02 | 0.0000 | 6024.0 | 6.00% | 3817.8 | 3.80% | motif file (matrix) | svg |
| 6 | C A G T T G C A A C G T A C T G C G T A A T C G C G A T T G A C C G T A A C G T | BATF(bZIP)/Th17-BATF-ChIP-Seq(GSE39756)/Homer | 1e-247 | -5.689e+02 | 0.0000 | 11460.0 | 11.41% | 8371.4 | 8.33% | motif file (matrix) | svg |
| 7 | C T A G T C G A G C A T C A T G G C T A T A G C C G A T G T A C C T G A A G C T | JunB(bZIP)/DendriticCells-Junb-ChIP-Seq(GSE36099)/Homer | 1e-243 | -5.617e+02 | 0.0000 | 9760.0 | 9.72% | 6929.3 | 6.90% | motif file (matrix) | svg |
| 8 | C T A G T C G A A C G T A C T G C G T A A T G C A C G T G T A C C G T A A G C T G A T C G T A C | Atf3(bZIP)/GBM-ATF3-ChIP-Seq(GSE33912)/Homer | 1e-228 | -5.253e+02 | 0.0000 | 11786.0 | 11.74% | 8766.5 | 8.73% | motif file (matrix) | svg |
| 9 | T C G A A C G T C A T G G C T A T A G C C G A T G T A C G C T A A C G T A T G C | AP-1(bZIP)/ThioMac-PU.1-ChIP-Seq(GSE21512)/Homer | 1e-215 | -4.960e+02 | 0.0000 | 12824.0 | 12.77% | 9759.6 | 9.72% | motif file (matrix) | svg |
| 10 | C G A T T A C G T G A C G A C T C A T G C G T A T A C G A C G T G T A C C T G A | Bach2(bZIP)/OCILy7-Bach2-ChIP-Seq(GSE44420)/Homer | 1e-136 | -3.147e+02 | 0.0000 | 3329.0 | 3.32% | 2104.7 | 2.10% | motif file (matrix) | svg |
| 11 | G A T C G T A C C G A T A C T G A C T G C G T A C G T A A C G T A C T G G A T C | TEAD(TEA)/Fibroblast-PU.1-ChIP-Seq(Unpublished)/Homer | 1e-129 | -2.973e+02 | 0.0000 | 12341.0 | 12.29% | 9975.4 | 9.93% | motif file (matrix) | svg |
| 12 | T G A C A G T C C T G A T G A C C G T A A C G T A C G T A G T C A G T C C G T A | TEAD1(TEAD)/HepG2-TEAD1-ChIP-Seq(Encode)/Homer | 1e-123 | -2.849e+02 | 0.0000 | 17378.0 | 17.31% | 14669.5 | 14.60% | motif file (matrix) | svg |
| 13 | T A G C T A G C G C A T C A T G A C T G G C T A C G T A A C G T A C T G G A T C | TEAD4(TEA)/Tropoblast-Tead4-ChIP-Seq(GSE37350)/Homer | 1e-122 | -2.822e+02 | 0.0000 | 13739.0 | 13.68% | 11312.1 | 11.26% | motif file (matrix) | svg |
| 14 | A G C T C T A G T G A C C G T A A C G T C G A T A G T C A G T C C T G A C A T G | TEAD3(TEA)/HepG2-TEAD3-ChIP-Seq(Encode)/Homer | 1e-112 | -2.586e+02 | 0.0000 | 19615.0 | 19.54% | 16894.2 | 16.82% | motif file (matrix) | svg |
| 15 | A T G C G A T C C G A T C T A G A C T G G C T A C G T A A G C T A C T G A G C T | TEAD2(TEA)/Py2T-Tead2-ChIP-Seq(GSE55709)/Homer | 1e-97 | -2.255e+02 | 0.0000 | 8618.0 | 8.58% | 6875.1 | 6.84% | motif file (matrix) | svg |
| 16 | A T G C G A C T A C G T C T A G A C G T A C G T A C G T C T G A G A T C G C T A A G C T C G T A | Foxa2(Forkhead)/Liver-Foxa2-ChIP-Seq(GSE25694)/Homer | 1e-96 | -2.231e+02 | 0.0000 | 13780.0 | 13.73% | 11610.5 | 11.56% | motif file (matrix) | svg |
| 17 | A T G C C T G A A T C G T A C G A G T C C G A T T C A G C G A T C T A G A G C T G T C A G T C A C G T A A G T C C G T A T A C G C T G A | Fox:Ebox(Forkhead,bHLH)/Panc1-Foxa2-ChIP-Seq(GSE47459)/Homer | 1e-96 | -2.229e+02 | 0.0000 | 15222.0 | 15.16% | 12953.7 | 12.90% | motif file (matrix) | svg |
| 18 | G C T A T C G A C G T A C T A G A G C T G T C A G T C A C G T A A G T C C G T A | FOXA1(Forkhead)/LNCAP-FOXA1-ChIP-Seq(GSE27824)/Homer | 1e-92 | -2.125e+02 | 0.0000 | 23023.0 | 22.93% | 20391.0 | 20.30% | motif file (matrix) | svg |
| 19 | C T A G G T A C A G T C T G C A A G T C C T G A A G T C A G T C A G T C G C T A | Klf4(Zf)/mES-Klf4-ChIP-Seq(GSE11431)/Homer | 1e-88 | -2.038e+02 | 0.0000 | 6364.0 | 6.34% | 4938.3 | 4.92% | motif file (matrix) | svg |
| 20 | G C T A T C G A C G T A C T A G A G C T G T C A G T C A C G T A A G T C C G T A | FOXA1(Forkhead)/MCF7-FOXA1-ChIP-Seq(GSE26831)/Homer | 1e-86 | -1.991e+02 | 0.0000 | 19932.0 | 19.85% | 17523.8 | 17.45% | motif file (matrix) | svg |
| 21 | A G T C T A G C G A C T A C G T C T A G A C G T A C G T A C G T C T G A A G T C G C T A G A C T C G T A C T A G A C T G | Foxa3(Forkhead)/Liver-Foxa3-ChIP-Seq(GSE77670)/Homer | 1e-85 | -1.973e+02 | 0.0000 | 6140.0 | 6.12% | 4761.6 | 4.74% | motif file (matrix) | svg |
| 22 | C G A T C T A G A C G T G T C A C G T A C G T A A G T C C G T A | Foxo3(Forkhead)/U2OS-Foxo3-ChIP-Seq(E-MTAB-2701)/Homer | 1e-83 | -1.919e+02 | 0.0000 | 12773.0 | 12.72% | 10826.0 | 10.78% | motif file (matrix) | svg |
| 23 | T A C G T C G A C A G T A C T G G C T A A T G C C G A T G T A C C G T A A C T G T A G C C G T A | NF-E2(bZIP)/K562-NFE2-ChIP-Seq(GSE31477)/Homer | 1e-83 | -1.913e+02 | 0.0000 | 1124.0 | 1.12% | 595.6 | 0.59% | motif file (matrix) | svg |
| 24 | G A C T T C A G C T A G A G T C A G T C G T A C A G T C C T G A A G T C A G T C A G T C G A C T A G T C A C T G A T G C | KLF3(Zf)/MEF-Klf3-ChIP-Seq(GSE44748)/Homer | 1e-80 | -1.843e+02 | 0.0000 | 6501.0 | 6.48% | 5126.3 | 5.10% | motif file (matrix) | svg |
| 25 | A G C T T G A C C G A T C G A T C T A G A C G T C A G T C A G T G C T A A G T C | FOXK1(Forkhead)/HEK293-FOXK1-ChIP-Seq(GSE51673)/Homer | 1e-78 | -1.810e+02 | 0.0000 | 17653.0 | 17.58% | 15468.1 | 15.40% | motif file (matrix) | svg |
| 26 | C G T A C G T A C G T A G C A T G C A T T A C G G T A C G A C T A C T G C G T A A T C G A C G T G T A C C G T A A G C T | Bach1(bZIP)/K562-Bach1-ChIP-Seq(GSE31477)/Homer | 1e-77 | -1.782e+02 | 0.0000 | 1176.0 | 1.17% | 649.0 | 0.65% | motif file (matrix) | svg |
| 27 | G T C A G C A T A C T G G T A C G A C T A C T G G C T A A T C G C A G T G T A C C G T A A G C T | Nrf2(bZIP)/Lymphoblast-Nrf2-ChIP-Seq(GSE37589)/Homer | 1e-73 | -1.698e+02 | 0.0000 | 1018.0 | 1.01% | 543.0 | 0.54% | motif file (matrix) | svg |
| 28 | A C G T C T A G A G C T A C G T A C G T C T G A A G T C G A C T A G C T C G T A | FOXM1(Forkhead)/MCF7-FOXM1-ChIP-Seq(GSE72977)/Homer | 1e-71 | -1.651e+02 | 0.0000 | 20036.0 | 19.96% | 17836.0 | 17.76% | motif file (matrix) | svg |
| 29 | A G T C C T A G C T A G A G T C G A T C G T A C A G T C C T A G A G T C A G T C A G T C G T A C | Sp2(Zf)/HEK293-Sp2.eGFP-ChIP-Seq(Encode)/Homer | 1e-68 | -1.585e+02 | 0.0000 | 19734.0 | 19.66% | 17591.1 | 17.51% | motif file (matrix) | svg |
| 30 | C G T A C T A G A C T G A C T G G A C T C T A G C A G T C T A G C A T G G A T C | KLF5(Zf)/LoVo-KLF5-ChIP-Seq(GSE49402)/Homer | 1e-66 | -1.539e+02 | 0.0000 | 18398.0 | 18.33% | 16346.1 | 16.27% | motif file (matrix) | svg |
| 31 | T C A G C T A G C T A G A C T G A C T G G A T C A C T G A C T G C T A G C T A G A G T C G A T C | KLF1(Zf)/HUDEP2-KLF1-CutnRun(GSE136251)/Homer | 1e-66 | -1.530e+02 | 0.0000 | 11926.0 | 11.88% | 10236.7 | 10.19% | motif file (matrix) | svg |
| 32 | A G C T A G T C A G T C A C G T C T A G A C G T A C G T A C G T C G T A A G T C G A T C C G T A | FOXP1(Forkhead)/H9-FOXP1-ChIP-Seq(GSE31006)/Homer | 1e-65 | -1.513e+02 | 0.0000 | 7601.0 | 7.57% | 6247.3 | 6.22% | motif file (matrix) | svg |
| 33 | C G T A G C T A C G A T C T A G A C G T G T C A C G T A C G T A A G T C C G T A T G C A T A C G | FoxL2(Forkhead)/Ovary-FoxL2-ChIP-Seq(GSE60858)/Homer | 1e-57 | -1.326e+02 | 0.0000 | 16086.0 | 16.02% | 14286.9 | 14.22% | motif file (matrix) | svg |
| 34 | G C A T G C A T C T G A A C G T C T G A A C G T C G T A C G T A C G T A A G T C G T C A G T C A | Foxf1(Forkhead)/Lung-Foxf1-ChIP-Seq(GSE77951)/Homer | 1e-53 | -1.235e+02 | 0.0000 | 17623.0 | 17.55% | 15817.7 | 15.75% | motif file (matrix) | svg |
| 35 | G C T A A T C G G C T A G A C T G C T A T C G A T A G C T C G A | GATA3(Zf)/iTreg-Gata3-ChIP-Seq(GSE20898)/Homer | 1e-51 | -1.184e+02 | 0.0000 | 24832.0 | 24.73% | 22811.7 | 22.71% | motif file (matrix) | svg |
| 36 | T A C G T A G C G C T A C G A T C T A G A C G T C A G T C A G T G C T A A G T C G T C A G C A T | FOXK2(Forkhead)/U2OS-FOXK2-ChIP-Seq(E-MTAB-2204)/Homer | 1e-50 | -1.169e+02 | 0.0000 | 10498.0 | 10.46% | 9103.7 | 9.06% | motif file (matrix) | svg |
| 37 | T G A C C G A T A C T G A C T G A C T G G A C T A C T G A C G T A C T G A C T G G A T C G A T C | EKLF(Zf)/Erythrocyte-Klf1-ChIP-Seq(GSE20478)/Homer | 1e-49 | -1.143e+02 | 0.0000 | 4147.0 | 4.13% | 3277.7 | 3.26% | motif file (matrix) | svg |
| 38 | A G C T A T G C G A C T G C A T C G T A A G C T G T A C C G A T A T C G A G T C | Gata6(Zf)/HUG1N-GATA6-ChIP-Seq(GSE51936)/Homer | 1e-48 | -1.115e+02 | 0.0000 | 15525.0 | 15.46% | 13899.4 | 13.84% | motif file (matrix) | svg |
| 39 | C G T A A C T G C G T A A C G T C G T A C T G A T C A G T G C A A G C T T G A C | TRPS1(Zf)/MCF7-TRPS1-ChIP-Seq(GSE107013)/Homer | 1e-47 | -1.083e+02 | 0.0000 | 33510.0 | 33.38% | 31387.3 | 31.25% | motif file (matrix) | svg |
| 40 | C G T A C G T A C G T A G C A T G C A T A C T G G T A C G A C T C T A G G C T A T A C G G A C T T G A C C G T A A G C T | NFE2L2(bZIP)/HepG2-NFE2L2-ChIP-Seq(Encode)/Homer | 1e-46 | -1.082e+02 | 0.0000 | 1264.0 | 1.26% | 819.3 | 0.82% | motif file (matrix) | svg |
| 41 | T C G A A G C T A C G T A C G T A G T C A G T C A C G T A T C G G A C T A T C G | EWS:ERG-fusion(ETS)/CADO\_ES1-EWS:ERG-ChIP-Seq(SRA014231)/Homer | 1e-45 | -1.038e+02 | 0.0000 | 11664.0 | 11.62% | 10283.7 | 10.24% | motif file (matrix) | svg |
| 42 | T A G C G C T A A C T G C G T A A C G T C G T A C G T A T A C G T C A G T C G A | Gata1(Zf)/K562-GATA1-ChIP-Seq(GSE18829)/Homer | 1e-43 | -9.980e+01 | 0.0000 | 9600.0 | 9.56% | 8363.3 | 8.33% | motif file (matrix) | svg |
| 43 | A G C T A G T C A T G C A G C T A C G T C G T A A C G T A G T C C G A T A T G C | Gata2(Zf)/K562-GATA2-ChIP-Seq(GSE18829)/Homer | 1e-43 | -9.941e+01 | 0.0000 | 10878.0 | 10.83% | 9569.7 | 9.53% | motif file (matrix) | svg |
| 44 | T A C G A T G C G C T A A C T G C G T A A C G T C G T A C T G A T A C G T C G A | Gata4(Zf)/Heart-Gata4-ChIP-Seq(GSE35151)/Homer | 1e-40 | -9.217e+01 | 0.0000 | 16801.0 | 16.73% | 15273.3 | 15.21% | motif file (matrix) | svg |
| 45 | T C G A T A G C G T C A A C T G A C T G C G T A C G T A C T A G A G C T T C A G | ERG(ETS)/VCaP-ERG-ChIP-Seq(GSE14097)/Homer | 1e-39 | -9.158e+01 | 0.0000 | 22338.0 | 22.25% | 20632.8 | 20.54% | motif file (matrix) | svg |
| 46 | G T A C C A G T A C T G A C T G A C T G G A T C A C T G A C G T A C T G A C T G A G T C G A T C | KLF6(Zf)/PDAC-KLF6-ChIP-Seq(GSE64557)/Homer | 1e-39 | -9.007e+01 | 0.0000 | 14807.0 | 14.75% | 13376.2 | 13.32% | motif file (matrix) | svg |
| 47 | T C A G T A G C G A C T C A T G C T G A A T C G G C A T G T A C C G T A A C T G T A G C T G C A | MafK(bZIP)/C2C12-MafK-ChIP-Seq(GSE36030)/Homer | 1e-37 | -8.708e+01 | 0.0000 | 3089.0 | 3.08% | 2434.6 | 2.42% | motif file (matrix) | svg |
| 48 | A G T C A C G T A C T G A G C T A C G T A C G T G T C A A G T C | Foxo1(Forkhead)/RAW-Foxo1-ChIP-Seq(Fan\_et\_al.)/Homer | 1e-34 | -7.960e+01 | 0.0000 | 27897.0 | 27.79% | 26181.3 | 26.06% | motif file (matrix) | svg |
| 49 | A T C G A G T C A G T C G A C T A T G C C T G A C T A G A C T G T A C G G T A C C T G A C G A T | AP-2gamma(AP2)/MCF7-TFAP2C-ChIP-Seq(GSE21234)/Homer | 1e-34 | -7.922e+01 | 0.0000 | 14732.0 | 14.67% | 13394.1 | 13.33% | motif file (matrix) | svg |
| 50 | C T A G T A C G A G T C C G T A A G T C A C G T A G T C T C G A C G T A T A C G | Nkx2.1(Homeobox)/LungAC-Nkx2.1-ChIP-Seq(GSE43252)/Homer | 1e-31 | -7.277e+01 | 0.0000 | 37616.0 | 37.47% | 35839.2 | 35.68% | motif file (matrix) | svg |
| 51 | A T G C A G T C C T G A A G T C C G A T A C G T A G T C A G T C A C G T A T C G G A C T A C G T | Etv2(ETS)/ES-ER71-ChIP-Seq(GSE59402)/Homer | 1e-31 | -7.155e+01 | 0.0000 | 12717.0 | 12.67% | 11526.9 | 11.48% | motif file (matrix) | svg |
| 52 | A G T C C T G A A G T C C G A T C A G T G A T C A T G C A C T G A T C G G A C T | Fli1(ETS)/CD8-FLI-ChIP-Seq(GSE20898)/Homer | 1e-30 | -7.027e+01 | 0.0000 | 14469.0 | 14.41% | 13220.9 | 13.16% | motif file (matrix) | svg |
| 53 | G C T A C G A T C A T G A T G C A G T C A G T C G A C T T A C G T C G A C T A G A C T G T A G C | AP-2alpha(AP2)/Hela-AP2alpha-ChIP-Seq(GSE31477)/Homer | 1e-30 | -6.926e+01 | 0.0000 | 11508.0 | 11.46% | 10388.6 | 10.34% | motif file (matrix) | svg |
| 54 | T G C A C T G A A T G C G T C A A C T G A C T G C G T A C G T A C T A G A G C T | Ets1-distal(ETS)/CD4+-PolII-ChIP-Seq(Barski\_et\_al.)/Homer | 1e-29 | -6.775e+01 | 0.0000 | 4170.0 | 4.15% | 3493.3 | 3.48% | motif file (matrix) | svg |
| 55 | C T G A C T G A C A T G A T C G A G C T A T C G G A C T C A T G C T G A G T C A | Tbr1(T-box)/Cortex-Tbr1-ChIP-Seq(GSE71384)/Homer | 1e-28 | -6.671e+01 | 0.0000 | 21179.0 | 21.10% | 19758.9 | 19.67% | motif file (matrix) | svg |
| 56 | C G A T C T G A C G T A C A G T A G T C G A T C G A T C A C T G | Pitx1(Homeobox)/Chicken-Pitx1-ChIP-Seq(GSE38910)/Homer | 1e-28 | -6.645e+01 | 0.0000 | 64185.0 | 63.93% | 62496.0 | 62.22% | motif file (matrix) | svg |
| 57 | G T A C G C T A T C A G C T G A C T A G C A T G A G C T G A T C T G C A T C G A C T G A A C T G C A G T A G T C G A T C G C T A | HNF4a(NR),DR1/HepG2-HNF4a-ChIP-Seq(GSE25021)/Homer | 1e-28 | -6.617e+01 | 0.0000 | 7052.0 | 7.02% | 6183.2 | 6.16% | motif file (matrix) | svg |
| 58 | T C G A T A G C T G C A A C T G A C T G C G T A C G T A C T A G G A C T T A C G | ETS1(ETS)/Jurkat-ETS1-ChIP-Seq(GSE17954)/Homer | 1e-28 | -6.547e+01 | 0.0000 | 13852.0 | 13.80% | 12670.5 | 12.61% | motif file (matrix) | svg |
| 59 | C T G A A C T G A C T G A G T C A G T C A G C T C T A G T A C G | ZFX(Zf)/mES-Zfx-ChIP-Seq(GSE11431)/Homer | 1e-28 | -6.463e+01 | 0.0000 | 16335.0 | 16.27% | 15075.7 | 15.01% | motif file (matrix) | svg |
| 60 | C G T A C T G A C T A G C T G A A G T C G C T A C G A T A T C G G A C T G A T C A G T C C T G A C T A G C T A G A G T C G C T A C G A T C T A G G A T C G A T C | p73(p53)/Trachea-p73-ChIP-Seq(PRJNA310161)/Homer | 1e-27 | -6.443e+01 | 0.0000 | 572.0 | 0.57% | 347.5 | 0.35% | motif file (matrix) | svg |
| 61 | A G C T G A C T C T A G C G T A C A T G C G A T C T A G A T C G G A C T C A G T | Bapx1(Homeobox)/VertebralCol-Bapx1-ChIP-Seq(GSE36672)/Homer | 1e-27 | -6.339e+01 | 0.0000 | 28608.0 | 28.49% | 27071.6 | 26.95% | motif file (matrix) | svg |
| 62 | C G T A C T A G C A T G A G C T A C T G C G A T A T C G C G T A G T C A G T C A | Tbet(T-box)/CD8-Tbet-ChIP-Seq(GSE33802)/Homer | 1e-27 | -6.309e+01 | 0.0000 | 15918.0 | 15.86% | 14687.8 | 14.62% | motif file (matrix) | svg |
| 63 | C G A T T A C G T G C A G T A C G A T C G A C T A G C T A C G T A T C G G T A C G A T C G T A C G A T C G T C A | PPARE(NR),DR1/3T3L1-Pparg-ChIP-Seq(GSE13511)/Homer | 1e-27 | -6.305e+01 | 0.0000 | 15685.0 | 15.62% | 14462.8 | 14.40% | motif file (matrix) | svg |
| 64 | C T G A T C A G C A G T C T A G A C T G C T A G G A T C A T C G A C T G C T G A T C A G G A T C | Sp5(Zf)/mES-Sp5.Flag-ChIP-Seq(GSE72989)/Homer | 1e-25 | -5.976e+01 | 0.0000 | 11589.0 | 11.54% | 10547.6 | 10.50% | motif file (matrix) | svg |
| 65 | C T G A T A G C T G A C T C A G C T A G G T C A C G T A T C A G A G C T T C A G | ETV4(ETS)/HepG2-ETV4-ChIP-Seq(ENCODE)/Homer | 1e-24 | -5.590e+01 | 0.0000 | 13485.0 | 13.43% | 12408.3 | 12.35% | motif file (matrix) | svg |
| 66 | A G C T A C G T A C T G A T G C A G T C C G T A C T G A T A C G | NF1-halfsite(CTF)/LNCaP-NF1-ChIP-Seq(Unpublished)/Homer | 1e-24 | -5.587e+01 | 0.0000 | 26070.0 | 25.97% | 24674.7 | 24.56% | motif file (matrix) | svg |
| 67 | A T G C G A C T A G C T C T A G C G T A C T A G C G A T C T A G A T C G G A T C | Nkx2.2(Homeobox)/NPC-Nkx2.2-ChIP-Seq(GSE61673)/Homer | 1e-23 | -5.503e+01 | 0.0000 | 25519.0 | 25.42% | 24144.9 | 24.04% | motif file (matrix) | svg |
| 68 | C T G A C T G A T A G C G A T C G C T A G T A C A C G T G A T C T G C A C G T A | Nkx2.5(Homeobox)/HL1-Nkx2.5.biotin-ChIP-Seq(GSE21529)/Homer | 1e-23 | -5.462e+01 | 0.0000 | 29977.0 | 29.86% | 28535.5 | 28.41% | motif file (matrix) | svg |
| 69 | G C T A G A C T G A C T T G C A C G T A A G T C C G T A T A G C G A T C G A C T | Eomes(T-box)/H9-Eomes-ChIP-Seq(GSE26097)/Homer | 1e-22 | -5.280e+01 | 0.0000 | 32320.0 | 32.19% | 30873.6 | 30.74% | motif file (matrix) | svg |
| 70 | T C G A T C G A T A C G G A T C G T C A G T A C C G A T A G C T G T C A T G C A | Nkx3.1(Homeobox)/LNCaP-Nkx3.1-ChIP-Seq(GSE28264)/Homer | 1e-21 | -5.061e+01 | 0.0000 | 32243.0 | 32.12% | 30829.4 | 30.69% | motif file (matrix) | svg |
| 71 | C T A G T C A G C A G T T C A G A C T G A C T G G A T C C T A G A C T G C T A G T C A G A T G C | KLF14(Zf)/HEK293-KLF14.GFP-ChIP-Seq(GSE58341)/Homer | 1e-21 | -4.982e+01 | 0.0000 | 22290.0 | 22.20% | 21046.1 | 20.95% | motif file (matrix) | svg |
| 72 | C G T A T A C G T C G A A C T G A C T G C G T A C G T A T A C G A G C T T A C G | PU.1(ETS)/ThioMac-PU.1-ChIP-Seq(GSE21512)/Homer | 1e-21 | -4.966e+01 | 0.0000 | 6855.0 | 6.83% | 6114.3 | 6.09% | motif file (matrix) | svg |
| 73 | C G T A T A G C T A G C T G C A A C T G C T A G C G T A C G T A T C A G G A C T | EHF(ETS)/LoVo-EHF-ChIP-Seq(GSE49402)/Homer | 1e-21 | -4.929e+01 | 0.0000 | 18919.0 | 18.84% | 17757.4 | 17.68% | motif file (matrix) | svg |
| 74 | A G C T C T G A C T A G C T A G A C T G T A G C T G C A T C G A C T G A C T A G C A T G A C G T A T G C T C G A | RXR(NR),DR1/3T3L1-RXR-ChIP-Seq(GSE13511)/Homer | 1e-20 | -4.672e+01 | 0.0000 | 18717.0 | 18.64% | 17592.5 | 17.51% | motif file (matrix) | svg |
| 75 | C G T A T G A C T A G C T G C A A C T G A C T G C G T A C G T A T C A G G A C T | ELF3(ETS)/PDAC-ELF3-ChIP-Seq(GSE64557)/Homer | 1e-19 | -4.574e+01 | 0.0000 | 11070.0 | 11.03% | 10181.7 | 10.14% | motif file (matrix) | svg |
| 76 | C G A T C T A G A C G T A C G T A C G T C G T A A G C T C G A T A G C T C G T A C T A G T A G C | FoxD3(forkhead)/ZebrafishEmbryo-Foxd3.biotin-ChIP-seq(GSE106676)/Homer | 1e-19 | -4.561e+01 | 0.0000 | 16328.0 | 16.26% | 15277.5 | 15.21% | motif file (matrix) | svg |
| 77 | G A T C T C G A A G T C C G A T C G A T A G T C A T G C A C T G A T C G G A C T | Elk1(ETS)/Hela-Elk1-ChIP-Seq(GSE31477)/Homer | 1e-18 | -4.359e+01 | 0.0000 | 6025.0 | 6.00% | 5374.7 | 5.35% | motif file (matrix) | svg |
| 78 | T G C A C T G A A G T C G T C A A C T G A C T G C G T A C G T A C T G A A G C T | EWS:FLI1-fusion(ETS)/SK\_N\_MC-EWS:FLI1-ChIP-Seq(SRA014231)/Homer | 1e-18 | -4.358e+01 | 0.0000 | 7128.0 | 7.10% | 6422.1 | 6.39% | motif file (matrix) | svg |
| 79 | A C G T C T A G C G T A A G T C G T A C A C G T A C G T A C G T G T C A G T A C T G A C G A C T | Nur77(NR)/K562-NR4A1-ChIP-Seq(GSE31363)/Homer | 1e-18 | -4.308e+01 | 0.0000 | 2867.0 | 2.86% | 2421.7 | 2.41% | motif file (matrix) | svg |
| 80 | T C G A C T G A T A G C T G A C T C A G T C A G C G T A C G T A T C A G A G C T | ETV1(ETS)/GIST48-ETV1-ChIP-Seq(GSE22441)/Homer | 1e-17 | -4.101e+01 | 0.0000 | 19355.0 | 19.28% | 18291.2 | 18.21% | motif file (matrix) | svg |
| 81 | T C A G T G A C G T A C T G C A G T A C C T A G G T A C A T G C A G T C G T C A A G T C G A C T | Klf9(Zf)/GBM-Klf9-ChIP-Seq(GSE62211)/Homer | 1e-17 | -4.079e+01 | 0.0000 | 5230.0 | 5.21% | 4643.7 | 4.62% | motif file (matrix) | svg |
| 82 | C G T A G A C T C G A T A T C G G T A C G C A T C A T G C G T A T A C G G C A T G T A C C G T A C A T G A T G C G C T A C T A G G C A T G C A T G C A T G A C T | MafB(bZIP)/BMM-Mafb-ChIP-Seq(GSE75722)/Homer | 1e-17 | -4.024e+01 | 0.0000 | 5700.0 | 5.68% | 5092.6 | 5.07% | motif file (matrix) | svg |
| 83 | G A C T C G A T T C A G G A T C G A C T A G C T A G C T A G T C G A T C C G T A C T A G C T A G T C G A T C G A C T G A | Bcl6(Zf)/Liver-Bcl6-ChIP-Seq(GSE31578)/Homer | 1e-16 | -3.826e+01 | 0.0000 | 20792.0 | 20.71% | 19738.9 | 19.65% | motif file (matrix) | svg |
| 84 | T G A C C G A T C T G A C T A G C T A G A C G T A T G C T G C A T C G A C T G A C T A G C A T G A C G T A G T C C G T A | PPARa(NR),DR1/Liver-Ppara-ChIP-Seq(GSE47954)/Homer | 1e-16 | -3.701e+01 | 0.0000 | 19134.0 | 19.06% | 18131.0 | 18.05% | motif file (matrix) | svg |
| 85 | C T G A C T G A C T G A A T G C G A T C C A T G A C T G G A C T G A C T G C A T C G T A C G T A A G T C G T A C C T G A A T C G G C A T G A C T G A C T A G C T | GRHL2(CP2)/HBE-GRHL2-ChIP-Seq(GSE46194)/Homer | 1e-16 | -3.698e+01 | 0.0000 | 5174.0 | 5.15% | 4619.5 | 4.60% | motif file (matrix) | svg |
| 86 | T G C A A G C T A C G T C T A G G A T C C T A G G A T C G T C A C T G A A G T C | CEBP(bZIP)/ThioMac-CEBPb-ChIP-Seq(GSE21512)/Homer | 1e-15 | -3.678e+01 | 0.0000 | 9852.0 | 9.81% | 9100.4 | 9.06% | motif file (matrix) | svg |
| 87 | T C A G C T G A C T A G C A T G A C G T A T G C C T G A C T G A C T G A C T A G C A T G A C G T A T G C C T G A | TR4(NR),DR1/Hela-TR4-ChIP-Seq(GSE24685)/Homer | 1e-15 | -3.557e+01 | 0.0000 | 1809.0 | 1.80% | 1490.6 | 1.48% | motif file (matrix) | svg |
| 88 | T A G C G T A C C G T A C T A G A C T G T G C A C G T A A T G C C G T A A T C G | AR-halfsite(NR)/LNCaP-AR-ChIP-Seq(GSE27824)/Homer | 1e-15 | -3.505e+01 | 0.0000 | 49519.0 | 49.32% | 48275.9 | 48.06% | motif file (matrix) | svg |
| 89 | C A T G A C G T A G T C G A T C G A T C G A T C G C T A C T A G C T A G C T A G T C A G T C G A | EBF1(EBF)/Near-E2A-ChIP-Seq(GSE21512)/Homer | 1e-15 | -3.482e+01 | 0.0000 | 16028.0 | 15.96% | 15124.9 | 15.06% | motif file (matrix) | svg |
| 90 | A G T C G A C T C A G T G T A C A G T C A T C G T C A G A C T G G T C A C G T A | Stat3(Stat)/mES-Stat3-ChIP-Seq(GSE11431)/Homer | 1e-14 | -3.436e+01 | 0.0000 | 6598.0 | 6.57% | 5997.6 | 5.97% | motif file (matrix) | svg |
| 91 | C A G T T C A G G A T C A C T G A C G T C T A G A C T G A C T G G A C T C T A G | Egr1(Zf)/K562-Egr1-ChIP-Seq(GSE32465)/Homer | 1e-14 | -3.401e+01 | 0.0000 | 7540.0 | 7.51% | 6903.1 | 6.87% | motif file (matrix) | svg |
| 92 | T G C A T C G A T A G C G T A C T C A G C T A G G T C A G C T A T C A G G A C T | ETS(ETS)/Promoter/Homer | 1e-14 | -3.389e+01 | 0.0000 | 3271.0 | 3.26% | 2849.3 | 2.84% | motif file (matrix) | svg |
| 93 | G A C T C T A G A T G C A G T C G T C A T A C G A T G C A T C G | HIC1(Zf)/Treg-ZBTB29-ChIP-Seq(GSE99889)/Homer | 1e-14 | -3.336e+01 | 0.0000 | 28551.0 | 28.44% | 27459.3 | 27.34% | motif file (matrix) | svg |
| 94 | C T G A C A G T C T G A A G T C C T A G G A C T A T C G G T A C | HIF-1b(HLH)/T47D-HIF1b-ChIP-Seq(GSE59937)/Homer | 1e-14 | -3.322e+01 | 0.0000 | 11329.0 | 11.28% | 10570.9 | 10.52% | motif file (matrix) | svg |
| 95 | C A T G G A C T T A C G G T C A G T A C G A T C G A C T A G C T A T C G T C G A T A C G T A G C | ERRg(NR)/Kidney-ESRRG-ChIP-Seq(GSE104905)/Homer | 1e-14 | -3.310e+01 | 0.0000 | 11889.0 | 11.84% | 11115.2 | 11.07% | motif file (matrix) | svg |
| 96 | C T A G C T G A T C A G A T C G A G C T C A T G G A C T A G T C C T G A T G C A | Tbx6(T-box)/ESC-Tbx6-ChIP-Seq(GSE93524)/Homer | 1e-14 | -3.254e+01 | 0.0000 | 15385.0 | 15.32% | 14528.1 | 14.46% | motif file (matrix) | svg |
| 97 | C T A G C A T G T G C A A C G T A G T C C G T A C A T G T C A G A C G T A C G T G C T A A G T C | Six1(Homeobox)/Myoblast-Six1-ChIP-Chip(GSE20150)/Homer | 1e-14 | -3.229e+01 | 0.0000 | 3669.0 | 3.65% | 3233.6 | 3.22% | motif file (matrix) | svg |
| 98 | G A C T C T A G G A T C C A G T A C T G C T G A A T G C G C A T A T G C C T G A | MafA(bZIP)/Islet-MafA-ChIP-Seq(GSE30298)/Homer | 1e-13 | -3.184e+01 | 0.0000 | 11329.0 | 11.28% | 10587.0 | 10.54% | motif file (matrix) | svg |
| 99 | A T G C T C G A A G T C A G C T A C G T G T A C A G T C G C T A C T A G C A T G G T C A C T G A T C A G A G T C | Stat3+il21(Stat)/CD4-Stat3-ChIP-Seq(GSE19198)/Homer | 1e-13 | -3.177e+01 | 0.0000 | 9520.0 | 9.48% | 8835.4 | 8.80% | motif file (matrix) | svg |
| 100 | T A G C T C A G C A T G G C A T A G C T C G A T A T G C C G T A C G T A G T C A | CHR(?)/Hela-CellCycle-Expression/Homer | 1e-13 | -3.126e+01 | 0.0000 | 11131.0 | 11.09% | 10402.4 | 10.36% | motif file (matrix) | svg |
| 101 | C A T G A G C T T A C G G T C A G T A C T A G C A G C T G A C T A T C G T C G A | Esrrb(NR)/mES-Esrrb-ChIP-Seq(GSE11431)/Homer | 1e-12 | -2.923e+01 | 0.0000 | 9643.0 | 9.60% | 8984.7 | 8.94% | motif file (matrix) | svg |
| 102 | G C T A A G T C T A C G T G C A A T C G T C A G G C T A T C G A T C A G A G C T | ELF5(ETS)/T47D-ELF5-ChIP-Seq(GSE30407)/Homer | 1e-12 | -2.871e+01 | 0.0000 | 10765.0 | 10.72% | 10079.8 | 10.03% | motif file (matrix) | svg |
| 103 | T A C G A C T G A G C T G T A C C G T A T C G A C T G A A C T G C A T G A C G T A G T C C G T A | COUP-TFII(NR)/K562-NR2F1-ChIP-Seq(Encode)/Homer | 1e-12 | -2.863e+01 | 0.0000 | 22185.0 | 22.10% | 21262.6 | 21.17% | motif file (matrix) | svg |
| 104 | A G T C G A C T C A G T A C T G C T A G T G A C G C T A A T G C G C A T A T C G C G A T A C T G G A T C G T A C G T C A C T G A | NF1(CTF)/LNCAP-NF1-ChIP-Seq(Unpublished)/Homer | 1e-12 | -2.835e+01 | 0.0000 | 5293.0 | 5.27% | 4805.8 | 4.78% | motif file (matrix) | svg |
| 105 | C T G A C T A G A C T G G C A T C T A G G C A T A T C G C T G A C G T A G T C A | Tbx21(T-box)/GM12878-TBX21-ChIP-Seq(Encode)/Homer | 1e-12 | -2.809e+01 | 0.0000 | 14581.0 | 14.52% | 13808.0 | 13.75% | motif file (matrix) | svg |
| 106 | C T A G C T A G A G T C T C A G A C T G A C G T A C G T C T G A | MYB(HTH)/ERMYB-Myb-ChIPSeq(GSE22095)/Homer | 1e-12 | -2.786e+01 | 0.0000 | 24145.0 | 24.05% | 23208.3 | 23.10% | motif file (matrix) | svg |
| 107 | C T A G C T G A A G T C G C T A C G A T A C T G G A C T G A T C G A T C C T G A C T A G C T G A T G A C G C T A C G A T T C A G G A C T G A T C G A T C T G A C | p53(p53)/Saos-p53-ChIP-Seq(GSE15780)/Homer | 1e-12 | -2.764e+01 | 0.0000 | 1090.0 | 1.09% | 875.5 | 0.87% | motif file (matrix) | svg |
| 108 | C T A G C T G A A G T C G C T A C G A T A C T G G A C T G A T C G A T C C T G A C T A G C T G A T G A C G C T A C G A T T C A G G A C T G A T C G A T C T G A C | p53(p53)/Saos-p53-ChIP-Seq/Homer | 1e-12 | -2.764e+01 | 0.0000 | 1090.0 | 1.09% | 875.5 | 0.87% | motif file (matrix) | svg |
| 109 | T A C G C T A G A T G C G A T C G T A C A G T C C T A G A G T C A G T C A G T C G T A C A G T C | Sp1(Zf)/Promoter/Homer | 1e-11 | -2.602e+01 | 0.0000 | 1878.0 | 1.87% | 1601.4 | 1.59% | motif file (matrix) | svg |
| 110 | T C G A T C G A T A G C G T A C T C A G T A C G C G T A C G T A T C A G A G C T | GABPA(ETS)/Jurkat-GABPa-ChIP-Seq(GSE17954)/Homer | 1e-11 | -2.595e+01 | 0.0000 | 11505.0 | 11.46% | 10836.0 | 10.79% | motif file (matrix) | svg |
| 111 | C G A T G A C T C G A T T C A G G A C T A C G T C A G T C T G A G A C T G A C T A G C T C G A T A C T G A T C G G T A C G C T A | NF1:FOXA1(CTF,Forkhead)/LNCAP-FOXA1-ChIP-Seq(GSE27824)/Homer | 1e-11 | -2.576e+01 | 0.0000 | 933.0 | 0.93% | 742.6 | 0.74% | motif file (matrix) | svg |
| 112 | C G T A C T G A A C T G A G C T A G T C G A T C G A T C G C A T C T G A C T A G C T A G T A C G T C G A T G C A G C A T | EBF2(EBF)/BrownAdipose-EBF2-ChIP-Seq(GSE97114)/Homer | 1e-10 | -2.522e+01 | 0.0000 | 14361.0 | 14.30% | 13636.1 | 13.58% | motif file (matrix) | svg |
| 113 | A G T C G C A T C G T A C G T A G T A C A C G T A C T G G A T C G A T C T C G A | BMYB(HTH)/Hela-BMYB-ChIP-Seq(GSE27030)/Homer | 1e-10 | -2.515e+01 | 0.0000 | 20971.0 | 20.89% | 20129.5 | 20.04% | motif file (matrix) | svg |
| 114 | G A T C C T G A A G T C C G A T C G A T G A T C A G T C A C T G A T C G A G C T | Elk4(ETS)/Hela-Elk4-ChIP-Seq(GSE31477)/Homer | 1e-10 | -2.487e+01 | 0.0000 | 6227.0 | 6.20% | 5735.9 | 5.71% | motif file (matrix) | svg |
| 115 | C T G A A G T C C G A T A G C T A T G C G T A C A C G T A T C G C A G T G C A T | Elf4(ETS)/BMDM-Elf4-ChIP-Seq(GSE88699)/Homer | 1e-10 | -2.468e+01 | 0.0000 | 14151.0 | 14.10% | 13439.2 | 13.38% | motif file (matrix) | svg |
| 116 | T C G A A C G T A C T G C T G A A G T C T C A G A G C T G T A C C G T A A G C T G A T C T C G A | JunD(bZIP)/K562-JunD-ChIP-Seq/Homer | 1e-10 | -2.453e+01 | 0.0000 | 978.0 | 0.97% | 787.4 | 0.78% | motif file (matrix) | svg |
| 117 | T G A C C T G A C T A G T C G A C T G A A T G C C G T A A C T G G C A T G T A C G C A T A T C G G C A T A G C T G A T C | PR(NR)/T47D-PR-ChIP-Seq(GSE31130)/Homer | 1e-10 | -2.387e+01 | 0.0000 | 28859.0 | 28.74% | 27948.5 | 27.82% | motif file (matrix) | svg |
| 118 | A T C G G A T C G C A T C G T A G T C A A C G T A T G C A G T C | CRX(Homeobox)/Retina-Crx-ChIP-Seq(GSE20012)/Homer | 1e-10 | -2.358e+01 | 0.0000 | 36809.0 | 36.66% | 35846.0 | 35.69% | motif file (matrix) | svg |
| 119 | G T C A T C G A C T A G C T A G A G T C G T C A C G A T C T A G G A C T G A T C G A T C T C A G C T A G C T G A A G T C G C T A C A G T T C A G G A T C G A T C | p63(p53)/Keratinocyte-p63-ChIP-Seq(GSE17611)/Homer | 1e-10 | -2.320e+01 | 0.0000 | 3691.0 | 3.68% | 3324.2 | 3.31% | motif file (matrix) | svg |
| 120 | G A C T G C A T C T A G C G A T G A T C T C G A C A T G G A T C | Tgif1(Homeobox)/mES-Tgif1-ChIP-Seq(GSE55404)/Homer | 1e-9 | -2.275e+01 | 0.0000 | 42199.0 | 42.03% | 41233.3 | 41.05% | motif file (matrix) | svg |
| 121 | T C A G G A C T C A G T C T G A A G C T C T A G G A C T T G C A C T G A A G T C | HLF(bZIP)/HSC-HLF.Flag-ChIP-Seq(GSE69817)/Homer | 1e-9 | -2.178e+01 | 0.0000 | 13440.0 | 13.39% | 12790.1 | 12.73% | motif file (matrix) | svg |
| 122 | C G T A A C G T A C T G G T A C C G T A A C G T C G T A C G T A A C G T A C T G A G T C C G T A A C G T C T G A G C A T | OCT:OCT-short(POU,Homeobox)/NPC-OCT6-ChIP-Seq(GSE43916)/Homer | 1e-9 | -2.139e+01 | 0.0000 | 12103.0 | 12.06% | 11488.2 | 11.44% | motif file (matrix) | svg |
| 123 | G A C T T C G A C G A T T A C G C T A G T A C G A C T G A T G C G T A C G T A C | Zac1(Zf)/Neuro2A-Plagl1-ChIP-Seq(GSE75942)/Homer | 1e-9 | -2.110e+01 | 0.0000 | 36742.0 | 36.60% | 35838.3 | 35.68% | motif file (matrix) | svg |
| 124 | T C A G T C A G A C G T G T A C G C T A T C A G C T G A A C T G A C T G A G C T A G T C C G T A | EAR2(NR)/K562-NR2F6-ChIP-Seq(Encode)/Homer | 1e-9 | -2.088e+01 | 0.0000 | 20238.0 | 20.16% | 19489.8 | 19.40% | motif file (matrix) | svg |
| 125 | A G C T A G C T C A T G C T G A G T A C A G T C A G C T A G C T C A G T C T A G | RARa(NR)/K562-RARa-ChIP-Seq(Encode)/Homer | 1e-8 | -1.953e+01 | 0.0000 | 43256.0 | 43.08% | 42368.4 | 42.18% | motif file (matrix) | svg |
| 126 | C T G A T A G C G C T A C G A T A T C G A G T C G A T C G A T C C T A G T C A G T C A G G T A C G C T A C A G T | p53(p53)/mES-cMyc-ChIP-Seq(GSE11431)/Homer | 1e-8 | -1.877e+01 | 0.0000 | 205.0 | 0.20% | 134.0 | 0.13% | motif file (matrix) | svg |
| 127 | A C T G C A T G G C T A T C G A G C T A A G C T A G C T G T A C A G T C T G A C | NFkB-p65-Rel(RHD)/ThioMac-LPS-Expression(GSE23622)/Homer | 1e-8 | -1.874e+01 | 0.0000 | 1483.0 | 1.48% | 1277.9 | 1.27% | motif file (matrix) | svg |
| 128 | C T G A C T A G A T C G A G C T A C T G G A C T A G T C C T G A | Tbx5(T-box)/HL1-Tbx5.biotin-ChIP-Seq(GSE21529)/Homer | 1e-8 | -1.870e+01 | 0.0000 | 44290.0 | 44.11% | 43422.2 | 43.23% | motif file (matrix) | svg |
| 129 | T C G A A C G T A C G T C T G A G A T C T C A G G A C T G T C A C G T A A G C T G T C A C T A G A G C T A C G T T C G A | NFIL3(bZIP)/HepG2-NFIL3-ChIP-Seq(Encode)/Homer | 1e-7 | -1.802e+01 | 0.0000 | 10203.0 | 10.16% | 9685.0 | 9.64% | motif file (matrix) | svg |
| 130 | G T A C G A T C C A G T A G T C A G T C A G T C T G C A G A T C C T G A A T G C G T C A A C G T | WT1(Zf)/Kidney-WT1-ChIP-Seq(GSE90016)/Homer | 1e-7 | -1.786e+01 | 0.0000 | 7517.0 | 7.49% | 7069.2 | 7.04% | motif file (matrix) | svg |
| 131 | G A C T A G T C C G A T A C T G C T G A T G A C G T A C C G T A A T C G G C A T C T G A C T A G | Bcl11a(Zf)/HSPC-BCL11A-ChIP-Seq(GSE104676)/Homer | 1e-7 | -1.771e+01 | 0.0000 | 10120.0 | 10.08% | 9609.6 | 9.57% | motif file (matrix) | svg |
| 132 | A C G T T A C G G A T C A C T G A C G T C T A G A C T G A C T G G A T C C T A G C A T G C T A G | Egr2(Zf)/Thymocytes-Egr2-ChIP-Seq(GSE34254)/Homer | 1e-7 | -1.756e+01 | 0.0000 | 1485.0 | 1.48% | 1286.4 | 1.28% | motif file (matrix) | svg |
| 133 | C G T A C T G A C G T A C T A G T C G A C T A G A C T G C G T A C G T A T A C G A G C T A T C G | SpiB(ETS)/OCILY3-SPIB-ChIP-Seq(GSE56857)/Homer | 1e-7 | -1.731e+01 | 0.0000 | 3442.0 | 3.43% | 3140.7 | 3.13% | motif file (matrix) | svg |
| 134 | T C A G G T C A G C T A A G T C C G T A A T C G T C G A T C A G C G T A A C T G A C T G A C T G | ZNF768(Zf)/Rajj-ZNF768-ChIP-Seq(GSE111879)/Homer | 1e-7 | -1.717e+01 | 0.0000 | 743.0 | 0.74% | 606.5 | 0.60% | motif file (matrix) | svg |
| 135 | G C A T C G A T C T A G G C T A G C A T C G T A G A T C G C T A C G A T C G A T C T A G C G A T C G T A C G A T G A T C | DMRT1(DM)/Testis-DMRT1-ChIP-Seq(GSE64892)/Homer | 1e-7 | -1.687e+01 | 0.0000 | 3427.0 | 3.41% | 3130.4 | 3.12% | motif file (matrix) | svg |
| 136 | T C A G T A C G T A G C A C G T A C T G C G A T A G T C C G T A T A C G A G T C | Meis1(Homeobox)/MastCells-Meis1-ChIP-Seq(GSE48085)/Homer | 1e-7 | -1.680e+01 | 0.0000 | 20670.0 | 20.59% | 20003.6 | 19.91% | motif file (matrix) | svg |
| 137 | C T G A C A T G A C T G A C G T A T G C C G T A C A T G T A C G A T G C G C T A T A C G C T G A C T A G A C T G A C G T A T G C C G T A T A G C | RAR:RXR(NR),DR5/ES-RAR-ChIP-Seq(GSE56893)/Homer | 1e-7 | -1.638e+01 | 0.0000 | 408.0 | 0.41% | 311.7 | 0.31% | motif file (matrix) | svg |
| 138 | T A G C G T A C A G T C G T A C C G A T A G T C A G T C A G T C A G T C A G T C C G T A G A T C | Zfp281(Zf)/ES-Zfp281-ChIP-Seq(GSE81042)/Homer | 1e-7 | -1.635e+01 | 0.0000 | 3197.0 | 3.18% | 2915.9 | 2.90% | motif file (matrix) | svg |
| 139 | C T A G G A T C T C G A A T G C T C G A A G C T C T G A A C T G A C G T C T G A A C T G A C T G A C G T A G T C G A T C | ZKSCAN1(Zf)/HepG2-ZKSCAN1-ChIP-Seq(Encode)/Homer | 1e-7 | -1.626e+01 | 0.0000 | 592.0 | 0.59% | 474.2 | 0.47% | motif file (matrix) | svg |
| 140 | G C A T A T C G C A T G G T A C G C T A A G T C T C A G T G A C G T C A T G C A | Arnt:Ahr(bHLH)/MCF7-Arnt-ChIP-Seq(Lo\_et\_al.)/Homer | 1e-6 | -1.610e+01 | 0.0000 | 6035.0 | 6.01% | 5654.9 | 5.63% | motif file (matrix) | svg |
| 141 | G T A C C G T A C G T A T A C G G C A T G T A C C G T A C A T G A G T C C G T A C G T A C G A T G C A T G C A T G A C T | MafF(bZIP)/HepG2-MafF-ChIP-Seq(GSE31477)/Homer | 1e-6 | -1.609e+01 | 0.0000 | 4510.0 | 4.49% | 4179.9 | 4.16% | motif file (matrix) | svg |
| 142 | A G T C C G A T A C T G A T C G T G A C G C T A C A T G A T C G T G A C C G A T A C T G T A G C G T A C G T C A | Tlx?(NR)/NPC-H3K4me1-ChIP-Seq(GSE16256)/Homer | 1e-6 | -1.609e+01 | 0.0000 | 5523.0 | 5.50% | 5158.9 | 5.14% | motif file (matrix) | svg |
| 143 | T C A G A G C T A C G T A C G T G T A C G A T C C G T A C T A G C A T G G T C A C G T A T C G A | STAT4(Stat)/CD4-Stat4-ChIP-Seq(GSE22104)/Homer | 1e-6 | -1.604e+01 | 0.0000 | 14401.0 | 14.34% | 13838.9 | 13.78% | motif file (matrix) | svg |
| 144 | C T G A A T G C G C T A C G A T A T G C C G T A C G T A C G T A C T A G T A C G | Tcf3(HMG)/mES-Tcf3-ChIP-Seq(GSE11724)/Homer | 1e-6 | -1.566e+01 | 0.0000 | 3754.0 | 3.74% | 3456.3 | 3.44% | motif file (matrix) | svg |
| 145 | T G A C C T A G A C T G T A G C C G A T A C T G A T G C C A T G A T C G A T C G A T C G T A G C C T G A T A G C G C T A A C T G C G T A A G C T C G T A C T G A | GATA:SCL(Zf,bHLH)/Ter119-SCL-ChIP-Seq(GSE18720)/Homer | 1e-6 | -1.543e+01 | 0.0000 | 1480.0 | 1.47% | 1295.0 | 1.29% | motif file (matrix) | svg |
| 146 | C T A G C A T G C A T G T A C G A G T C G C A T A G C T C T A G A C G T A G T C G A C T A C T G A C T G A C T G T C G A | Zfp809(Zf)/ES-Zfp809-ChIP-Seq(GSE70799)/Homer | 1e-6 | -1.537e+01 | 0.0000 | 2868.0 | 2.86% | 2610.3 | 2.60% | motif file (matrix) | svg |
| 147 | C T A G C G T A G T C A C G T A A G T C G A T C A G C T C T A G C G T A A C G T G T C A G A T C | Six2(Homeobox)/NephronProgenitor-Six2-ChIP-Seq(GSE39837)/Homer | 1e-6 | -1.496e+01 | 0.0000 | 15149.0 | 15.09% | 14597.4 | 14.53% | motif file (matrix) | svg |
| 148 | A G T C C G A T C T G A C G T A A C G T C A G T T C A G T G A C | Isl1(Homeobox)/Neuron-Isl1-ChIP-Seq(GSE31456)/Homer | 1e-6 | -1.489e+01 | 0.0000 | 31287.0 | 31.16% | 30576.8 | 30.44% | motif file (matrix) | svg |
| 149 | C G T A C G A T G A C T G A C T T G A C C T G A A T G C C T G A T A G C A G T C A C G T T C G A C A T G T A C G G A C T A T C G G A C T A C G T C T G A T C G A C G T A | Brachyury(T-box)/Mesoendoderm-Brachyury-ChIP-exo(GSE54963)/Homer | 1e-6 | -1.388e+01 | 0.0000 | 3890.0 | 3.87% | 3607.0 | 3.59% | motif file (matrix) | svg |
| 150 | G C T A A C G T C T A G G T A C G C T A G A C T C T G A G C A T C A T G G A T C | Pit1(Homeobox)/GCrat-Pit1-ChIP-Seq(GSE58009)/Homer | 1e-6 | -1.384e+01 | 0.0000 | 18151.0 | 18.08% | 17585.0 | 17.51% | motif file (matrix) | svg |
| 151 | C T G A T G C A T A G C T G A C T A C G T C A G C T G A G C T A T C A G G A C T | ELF1(ETS)/Jurkat-ELF1-ChIP-Seq(SRA014231)/Homer | 1e-5 | -1.378e+01 | 0.0000 | 5253.0 | 5.23% | 4927.4 | 4.91% | motif file (matrix) | svg |
| 152 | A G T C G A C T A C T G G A T C G T A C C G T A T G A C A G T C C G A T A G C T A C G T A C G T C T A G G A C T C T G A | ZNF7(Zf)/HepG2-ZNF7.Flag-ChIP-Seq(Encode)/Homer | 1e-5 | -1.375e+01 | 0.0000 | 7699.0 | 7.67% | 7309.2 | 7.28% | motif file (matrix) | svg |
| 153 | T G A C T C G A C T G A C T G A A T G C G A T C C T A G T A C G G A C T G A C T G A T C T C G A C T G A C T G A A T G C G A T C C T A G A T C G G A C T G A C T | Tcfcp2l1(CP2)/mES-Tcfcp2l1-ChIP-Seq(GSE11431)/Homer | 1e-5 | -1.354e+01 | 0.0000 | 1497.0 | 1.49% | 1324.8 | 1.32% | motif file (matrix) | svg |
| 154 | C T G A C T G A C T A G T C G A C G T A A T G C C G T A A C T G C G T A A C G T C T G A C G A T A G C T C G T A A C G T A G T C C G A T T A C G G T C A G C A T | GATA(Zf),IR3/iTreg-Gata3-ChIP-Seq(GSE20898)/Homer | 1e-5 | -1.342e+01 | 0.0000 | 2239.0 | 2.23% | 2028.1 | 2.02% | motif file (matrix) | svg |
| 155 | T A C G C T G A T C G A C G A T C T A G C T A G T C G A C T G A T C G A T C G A C G T A T C G A G C A T C A T G C G T A T A C G G C A T T G A C C G T A A G C T | NFAT:AP1(RHD,bZIP)/Jurkat-NFATC1-ChIP-Seq(Jolma\_et\_al.)/Homer | 1e-5 | -1.339e+01 | 0.0000 | 2855.0 | 2.84% | 2617.2 | 2.61% | motif file (matrix) | svg |
| 156 | C A T G T G A C C G T A A G T C T A C G G C A T A C T G G T C A A T G C A G T C | bHLHE41(bHLH)/proB-Bhlhe41-ChIP-Seq(GSE93764)/Homer | 1e-5 | -1.315e+01 | 0.0000 | 11254.0 | 11.21% | 10803.3 | 10.76% | motif file (matrix) | svg |
| 157 | A T G C C T G A G A C T A C G T A C G T G T A C G A T C C G A T C T A G C A T G C G T A C G T A C T G A G A C T | STAT1(Stat)/HelaS3-STAT1-ChIP-Seq(GSE12782)/Homer | 1e-5 | -1.271e+01 | 0.0000 | 4414.0 | 4.40% | 4128.0 | 4.11% | motif file (matrix) | svg |
| 158 | T G C A A C T G T A C G A T G C A G T C G A C T T C G A A T C G | ZNF711(Zf)/SHSY5Y-ZNF711-ChIP-Seq(GSE20673)/Homer | 1e-5 | -1.246e+01 | 0.0000 | 24066.0 | 23.97% | 23476.5 | 23.37% | motif file (matrix) | svg |
| 159 | C T A G C A T G C A T G A G C T G A C T C G T A G A T C A G C T G T C A A T G C T C G A C T A G C A T G A C G T A G T C C T G A | LXRE(NR),DR4/RAW-LXRb.biotin-ChIP-Seq(GSE21512)/Homer | 1e-5 | -1.242e+01 | 0.0000 | 627.0 | 0.62% | 522.5 | 0.52% | motif file (matrix) | svg |
| 160 | A T G C A G T C A G C T A G C T A C G T A T C G C G T A C G A T T A G C G A C T | LEF1(HMG)/H1-LEF1-ChIP-Seq(GSE64758)/Homer | 1e-5 | -1.179e+01 | 0.0000 | 10558.0 | 10.52% | 10147.0 | 10.10% | motif file (matrix) | svg |
| 161 | C T A G A G C T G A C T C A T G A G T C A G T C G T C A C A G T C T A G T C A G G T A C C T G A T C G A G A T C T G A C | Rfx2(HTH)/LoVo-RFX2-ChIP-Seq(GSE49402)/Homer | 1e-5 | -1.175e+01 | 0.0000 | 1020.0 | 1.02% | 890.0 | 0.89% | motif file (matrix) | svg |
| 162 | T C A G C T A G C T A G T G C A C G A T C G A T C G T A C T A G | GSC(Homeobox)/FrogEmbryos-GSC-ChIP-Seq(DRA000576)/Homer | 1e-4 | -1.144e+01 | 0.0000 | 19427.0 | 19.35% | 18908.7 | 18.82% | motif file (matrix) | svg |
| 163 | T A C G T A C G G T A C A T C G T A C G T A C G G T C A C T G A C G T A G A C T | E2F4(E2F)/K562-E2F4-ChIP-Seq(GSE31477)/Homer | 1e-4 | -1.128e+01 | 0.0000 | 3234.0 | 3.22% | 3005.9 | 2.99% | motif file (matrix) | svg |
| 164 | C T A G C A T G A C G T A G T C G C T A A G C T A G T C A G C T T C A G C T G A A C T G C A T G G C A T A T G C C G T A | THRa(NR)/C17.2-THRa-ChIP-Seq(GSE38347)/Homer | 1e-4 | -1.118e+01 | 0.0000 | 5091.0 | 5.07% | 4807.1 | 4.79% | motif file (matrix) | svg |
| 165 | A T G C A G T C G C T A C G A T C G T A G C A T G C T A C G A T C T A G C A T G T G A C G T C A | CArG(MADS)/PUER-Srf-ChIP-Seq(Sullivan\_et\_al.)/Homer | 1e-4 | -1.115e+01 | 0.0000 | 5185.0 | 5.16% | 4899.5 | 4.88% | motif file (matrix) | svg |
| 166 | T A C G C T G A C T A G T C G A C G T A A G T C C T G A A T C G G C A T T A G C G A C T A C T G A C G T A G C T A G T C G A C T | GRE(NR),IR3/A549-GR-ChIP-Seq(GSE32465)/Homer | 1e-4 | -1.084e+01 | 0.0001 | 1514.0 | 1.51% | 1361.6 | 1.36% | motif file (matrix) | svg |
| 167 | C G A T A C G T A C G T A C G T C G T A A G C T C A G T C T A G A T C G A C T G | HOXB13(Homeobox)/ProstateTumor-HOXB13-ChIP-Seq(GSE56288)/Homer | 1e-4 | -1.073e+01 | 0.0001 | 19206.0 | 19.13% | 18710.0 | 18.63% | motif file (matrix) | svg |
| 168 | T C G A T G C A A C T G G T C A A G T C A G T C A G T C A G T C A G C T T G A C | LRF(Zf)/Erythroblasts-ZBTB7A-ChIP-Seq(GSE74977)/Homer | 1e-4 | -1.046e+01 | 0.0001 | 18677.0 | 18.60% | 18193.9 | 18.11% | motif file (matrix) | svg |
| 169 | G A C T C G A T C A T G G C T A G C A T C G T A A G T C G C T A G C A T C G A T T A C G G C A T C G T A C A G T G A T C | DMRT6(DM)/Testis-DMRT6-ChIP-Seq(GSE60440)/Homer | 1e-4 | -9.882e+00 | 0.0001 | 2658.0 | 2.65% | 2466.8 | 2.46% | motif file (matrix) | svg |
| 170 | C G T A C G T A C G T A A G T C A G C T C T G A A C T G A C T G A G C T A G T C C G T A C T A G C T A G C T A G T G C A | RORa(NR)/Liver-Rora-ChIP-Seq(GSE101115)/Homer | 1e-4 | -9.874e+00 | 0.0001 | 2259.0 | 2.25% | 2082.7 | 2.07% | motif file (matrix) | svg |
| 171 | T A G C C G A T T A C G A C T G A G T C A C T G A T C G A T C G C G T A C T G A | E2F1(E2F)/Hela-E2F1-ChIP-Seq(GSE22478)/Homer | 1e-4 | -9.714e+00 | 0.0002 | 1570.0 | 1.56% | 1424.7 | 1.42% | motif file (matrix) | svg |
| 172 | T C A G G C T A T C A G C A G T T G A C G T C A A G T C A T C G T G C A G T A C C A G T G A T C | Npas4(bHLH)/Neuron-Npas4-ChIP-Seq(GSE127793)/Homer | 1e-4 | -9.628e+00 | 0.0002 | 8193.0 | 8.16% | 7869.2 | 7.83% | motif file (matrix) | svg |
| 173 | G C T A G C A T G A C T G C A T T C A G G T A C G C T A G C A T C T G A G C T A T A G C G C T A C T G A C G A T C T A G | OCT4-SOX2-TCF-NANOG(POU,Homeobox,HMG)/mES-Oct4-ChIP-Seq(GSE11431)/Homer | 1e-4 | -9.583e+00 | 0.0002 | 3029.0 | 3.02% | 2828.8 | 2.82% | motif file (matrix) | svg |
| 174 | A T G C T A C G A G C T T G C A C G T A C G A T A C G T C T G A | DLX5(Homeobox)/BasalGanglia-Dlx5-ChIP-seq(GSE124936)/Homer | 1e-4 | -9.569e+00 | 0.0002 | 13579.0 | 13.53% | 13176.7 | 13.12% | motif file (matrix) | svg |
| 175 | A C G T G A C T T A G C C G T A C T G A C A T G C T A G G A C T G A T C C G T A | Nr5a2(NR)/Pancreas-LRH1-ChIP-Seq(GSE34295)/Homer | 1e-4 | -9.416e+00 | 0.0002 | 9965.0 | 9.93% | 9616.6 | 9.57% | motif file (matrix) | svg |
| 176 | T G C A G T A C C G T A A T C G A C T G A C G T C T A G C G A T T C G A A G T C | ZEB1(Zf)/PDAC-ZEB1-ChIP-Seq(GSE64557)/Homer | 1e-4 | -9.299e+00 | 0.0002 | 25553.0 | 25.45% | 25051.8 | 24.94% | motif file (matrix) | svg |
| 177 | C T A G C T A G G C A T C G A T C T G A G T C A G C T A A T G C C G T A C A G T G A C T C G T A T G C A | Hnf1(Homeobox)/Liver-Foxa2-Chip-Seq(GSE25694)/Homer | 1e-4 | -9.241e+00 | 0.0002 | 2767.0 | 2.76% | 2579.4 | 2.57% | motif file (matrix) | svg |
| 178 | C T A G A C G T G C A T T C G A G T C A G C A T A T C G C G T A C A G T A G C T C T G A T G C A | HNF1b(Homeobox)/PDAC-HNF1B-ChIP-Seq(GSE64557)/Homer | 1e-3 | -9.182e+00 | 0.0003 | 3067.0 | 3.05% | 2870.2 | 2.86% | motif file (matrix) | svg |
| 179 | C A T G A C T G A G C T A T G C C G T A A T G C G T A C G A C T T A C G C T G A A C T G A C T G G C A T A T G C C T G A | THRb(NR)/HepG2-THRb.Flag-ChIP-Seq(Encode)/Homer | 1e-3 | -9.142e+00 | 0.0003 | 6908.0 | 6.88% | 6618.6 | 6.59% | motif file (matrix) | svg |
| 180 | T A C G C T G A C A T G G A T C G T A C G C A T T C A G T A C G A G C T G T C A G A T C G C A T T A C G C G T A C T A G G A T C G A T C C G A T A C T G T C A G | ZNF322(Zf)/HEK293-ZNF322.GFP-ChIP-Seq(GSE58341)/Homer | 1e-3 | -9.072e+00 | 0.0003 | 4052.0 | 4.04% | 3828.9 | 3.81% | motif file (matrix) | svg |
| 181 | T A G C G C A T A G T C G A T C A T G C G A C T C T A G A C T G A C T G C T G A A C T G C T A G A G T C T G A C C G A T | GLIS3(Zf)/Thyroid-Glis3.GFP-ChIP-Seq(GSE103297)/Homer | 1e-3 | -9.065e+00 | 0.0003 | 19772.0 | 19.69% | 19320.4 | 19.23% | motif file (matrix) | svg |
| 182 | C A T G G A C T G C A T A C T G A G C T A C T G A C T G C G T A G C A T A G C T A T C G T A C G | Foxh1(Forkhead)/hESC-FOXH1-ChIP-Seq(GSE29422)/Homer | 1e-3 | -9.015e+00 | 0.0003 | 9924.0 | 9.88% | 9586.0 | 9.54% | motif file (matrix) | svg |
| 183 | C T G A C T A G A T C G G C A T A C T G G T A C A T G C C G T A A C T G G C T A A G T C C G T A | Tbox:Smad(T-box,MAD)/ESCd5-Smad2\_3-ChIP-Seq(GSE29422)/Homer | 1e-3 | -8.988e+00 | 0.0003 | 2148.0 | 2.14% | 1985.5 | 1.98% | motif file (matrix) | svg |
| 184 | A T G C C G T A A C T G C G T A A C G T G C T A T C G A A G C T C G A T C G T A A C G T A G T C C G A T A C T G G A T C | GATA(Zf),IR4/iTreg-Gata3-ChIP-Seq(GSE20898)/Homer | 1e-3 | -8.963e+00 | 0.0003 | 1252.0 | 1.25% | 1128.6 | 1.12% | motif file (matrix) | svg |
| 185 | A C G T G A C T A T G C G C T A C T G A C T A G A C T G G A C T A G T C C G T A | Nr5a2(NR)/mES-Nr5a2-ChIP-Seq(GSE19019)/Homer | 1e-3 | -8.932e+00 | 0.0003 | 7342.0 | 7.31% | 7048.3 | 7.02% | motif file (matrix) | svg |
| 186 | C A T G A G T C A G C T C G T A C G A T C G A T G C A T G C A T C G A T C T G A C A T G T G A C | Mef2d(MADS)/Retina-Mef2d-ChIP-Seq(GSE61391)/Homer | 1e-3 | -8.902e+00 | 0.0003 | 3494.0 | 3.48% | 3288.8 | 3.27% | motif file (matrix) | svg |
| 187 | T C A G C A T G C A T G A C T G A C T G A G C T A C T G A C G T A C T G C A G T A T G C A G T C | KLF10(Zf)/HEK293-KLF10.GFP-ChIP-Seq(GSE58341)/Homer | 1e-3 | -8.794e+00 | 0.0004 | 8067.0 | 8.04% | 7763.4 | 7.73% | motif file (matrix) | svg |
| 188 | C T G A C T A G A C T G G C A T A T G C C G T A C T G A C T A G A C T G A C G T A G T C C T G A | RARg(NR)/ES-RARg-ChIP-Seq(GSE30538)/Homer | 1e-3 | -8.762e+00 | 0.0004 | 448.0 | 0.45% | 376.2 | 0.37% | motif file (matrix) | svg |
| 189 | C G T A A T G C C G A T A C G T A G T C C G T A C G T A C G T A C T A G A T C G | TCFL2(HMG)/K562-TCF7L2-ChIP-Seq(GSE29196)/Homer | 1e-3 | -8.595e+00 | 0.0004 | 1116.0 | 1.11% | 1002.9 | 1.00% | motif file (matrix) | svg |
| 190 | C T G A T C A G C T G A C T A G C A T G A C G T A T G C C G T A A T G C G C A T T C A G C T G A A C T G A C G T C A G T A G T C C G T A C A G T C T A G C A T G | VDR(NR),DR3/GM10855-VDR+vitD-ChIP-Seq(GSE22484)/Homer | 1e-3 | -8.547e+00 | 0.0004 | 2826.0 | 2.81% | 2645.3 | 2.63% | motif file (matrix) | svg |
| 191 | T G A C C T G A C T A G C T G A C G T A A G T C C T G A A C G T G C A T T A G C G C A T A T C G G A C T G A C T G A T C | GRE(NR),IR3/RAW264.7-GRE-ChIP-Seq(Unpublished)/Homer | 1e-3 | -8.546e+00 | 0.0004 | 2701.0 | 2.69% | 2524.2 | 2.51% | motif file (matrix) | svg |
| 192 | C T G A A T G C C G T A A C G T A G T C A G T C A C G T A C T G A T C G G C A T | SPDEF(ETS)/VCaP-SPDEF-ChIP-Seq(SRA014231)/Homer | 1e-3 | -8.463e+00 | 0.0005 | 16961.0 | 16.89% | 16553.6 | 16.48% | motif file (matrix) | svg |
| 193 | A T G C T C A G T C G A G C A T A C T G C G T A A G T C T C A G G A C T T G A C C G T A A G C T | Atf2(bZIP)/3T3L1-Atf2-ChIP-Seq(GSE56872)/Homer | 1e-3 | -8.278e+00 | 0.0006 | 3875.0 | 3.86% | 3668.3 | 3.65% | motif file (matrix) | svg |
| 194 | A G C T C A T G G C A T G A T C T G C A C T A G G A T C A C G T | Tgif2(Homeobox)/mES-Tgif2-ChIP-Seq(GSE55404)/Homer | 1e-3 | -8.128e+00 | 0.0007 | 46065.0 | 45.88% | 45545.7 | 45.34% | motif file (matrix) | svg |
| 195 | T C G A T A G C T G A C C T G A A G T C A C T G G A C T C A T G | c-Myc(bHLH)/LNCAP-cMyc-ChIP-Seq(Unpublished)/Homer | 1e-3 | -8.063e+00 | 0.0007 | 4298.0 | 4.28% | 4084.7 | 4.07% | motif file (matrix) | svg |
| 196 | C T A G A C T G C T A G T C A G T C A G T A C G C T A G A C T G | Maz(Zf)/HepG2-Maz-ChIP-Seq(GSE31477)/Homer | 1e-3 | -8.037e+00 | 0.0007 | 17797.0 | 17.73% | 17395.0 | 17.32% | motif file (matrix) | svg |
| 197 | T A C G T C A G A G C T A T G C C G T A A G T C T C A G A C G T A C T G T C G A | USF1(bHLH)/GM12878-Usf1-ChIP-Seq(GSE32465)/Homer | 1e-3 | -7.969e+00 | 0.0008 | 4937.0 | 4.92% | 4710.5 | 4.69% | motif file (matrix) | svg |
| 198 | A C T G A C G T C A T G A T C G A T C G T G A C A C T G A T C G A T C G T G C A C T G A C G T A | E2F3(E2F)/MEF-E2F3-ChIP-Seq(GSE71376)/Homer | 1e-3 | -7.924e+00 | 0.0008 | 5448.0 | 5.43% | 5211.6 | 5.19% | motif file (matrix) | svg |
| 199 | G A C T C T A G C T A G A G T C T G C A A C T G A C G T A C G T C T A G T C A G | AMYB(HTH)/Testes-AMYB-ChIP-Seq(GSE44588)/Homer | 1e-3 | -7.832e+00 | 0.0009 | 20677.0 | 20.60% | 20259.1 | 20.17% | motif file (matrix) | svg |
| 200 | G T C A T G C A G C T A A G T C C G T A A C T G T G A C G C A T T C A G C A G T | Ap4(bHLH)/AML-Tfap4-ChIP-Seq(GSE45738)/Homer | 1e-3 | -7.784e+00 | 0.0009 | 18073.0 | 18.00% | 17677.2 | 17.60% | motif file (matrix) | svg |
| 201 | T C G A C T G A C T G A A T G C G T A C G T C A A G C T A G C T C G T A T C A G | Barx1(Homeobox)/Stomach-Barx1.3xFlag-ChIP-Seq(GSE69483)/Homer | 1e-3 | -7.776e+00 | 0.0009 | 8451.0 | 8.42% | 8164.8 | 8.13% | motif file (matrix) | svg |
| 202 | A G T C A T C G C T A G A G C T G A C T C T A G A G T C A G T C G C T A C A G T T C A G T C A G G A T C C T G A T C G A G A T C | RFX(HTH)/K562-RFX3-ChIP-Seq(SRA012198)/Homer | 1e-3 | -7.653e+00 | 0.0010 | 826.0 | 0.82% | 735.8 | 0.73% | motif file (matrix) | svg |
| 203 | T G A C G T C A A G T C G C T A C T A G A G T C C T G A C A T G A C T G C T A G T A C G T C A G | Zic2(Zf)/ESC-Zic2-ChIP-Seq(SRP197560)/Homer | 1e-3 | -7.628e+00 | 0.0011 | 6431.0 | 6.41% | 6181.0 | 6.15% | motif file (matrix) | svg |
| 204 | T C G A A C T G C A T G A G C T A G T C C G T A C T G A C T A G A C T G C G A T A T G C C T G A | RAR:RXR(NR),DR0/ES-RAR-ChIP-Seq(GSE56893)/Homer | 1e-3 | -7.452e+00 | 0.0013 | 1675.0 | 1.67% | 1547.6 | 1.54% | motif file (matrix) | svg |
| 205 | T G A C G C T A T G A C C G T A T C A G G A T C C G T A C A T G C A T G C T A G C T A G C T A G | Unknown-ESC-element(?)/mES-Nanog-ChIP-Seq(GSE11724)/Homer | 1e-3 | -7.272e+00 | 0.0015 | 8959.0 | 8.92% | 8677.4 | 8.64% | motif file (matrix) | svg |
| 206 | T C G A T G A C A G T C C G T A A G T C C T A G A C G T A C T G A C T G A G C T A G T C G C A T | Max(bHLH)/K562-Max-ChIP-Seq(GSE31477)/Homer | 1e-3 | -7.191e+00 | 0.0016 | 7548.0 | 7.52% | 7289.3 | 7.26% | motif file (matrix) | svg |
| 207 | C G T A A C G T A G C T C G A T C T A G G T A C C G T A A G C T C G T A G C T A | Oct4(POU,Homeobox)/mES-Oct4-ChIP-Seq(GSE11431)/Homer | 1e-3 | -7.187e+00 | 0.0016 | 7682.0 | 7.65% | 7421.7 | 7.39% | motif file (matrix) | svg |
| 208 | T C A G A G C T A T G C C G T A A G C T T C A G C A G T A C T G C T G A A G T C | MITF(bHLH)/MastCells-MITF-ChIP-Seq(GSE48085)/Homer | 1e-3 | -7.184e+00 | 0.0016 | 11227.0 | 11.18% | 10918.3 | 10.87% | motif file (matrix) | svg |
| 209 | T C G A C A T G C A T G T A C G G T A C T C G A A G T C C A G T C T G A C G T A A G T C G A C T | ZNF264(Zf)/HEK293-ZNF264.GFP-ChIP-Seq(GSE58341)/Homer | 1e-3 | -7.175e+00 | 0.0016 | 7895.0 | 7.86% | 7631.9 | 7.60% | motif file (matrix) | svg |
| 210 | C T A G C T A G C G T A C G T A T A C G C G A T C T A G C T G A C T G A C G T A T A C G G A C T | PU.1:IRF8(ETS:IRF)/pDC-Irf8-ChIP-Seq(GSE66899)/Homer | 1e-3 | -7.062e+00 | 0.0018 | 2853.0 | 2.84% | 2693.0 | 2.68% | motif file (matrix) | svg |
| 211 | C G T A C T A G G A C T G T C A G T C A C G T A A G T C C G T A T C G A T C G A T C G A C G T A C T G A C T A G G C T A C G T A T A G C C G T A C G A T C G T A | FOXA1:AR(Forkhead,NR)/LNCAP-AR-ChIP-Seq(GSE27824)/Homer | 1e-3 | -7.010e+00 | 0.0019 | 1388.0 | 1.38% | 1276.4 | 1.27% | motif file (matrix) | svg |
| 212 | A G T C G A T C G C T A C G A T A C G T T A C G G C A T C T G A G A C T A C T G A G T C G C T A C T G A T C G A C A G T | Oct4:Sox17(POU,Homeobox,HMG)/F9-Sox17-ChIP-Seq(GSE44553)/Homer | 1e-3 | -6.955e+00 | 0.0020 | 2007.0 | 2.00% | 1873.1 | 1.86% | motif file (matrix) | svg |
| 213 | C T A G T C G A C G A T C T A G G C A T C A G T C T A G G A T C C G T A G T C A | CEBP:AP1(bZIP)/ThioMac-CEBPb-ChIP-Seq(GSE21512)/Homer | 1e-2 | -6.789e+00 | 0.0023 | 11178.0 | 11.13% | 10881.7 | 10.83% | motif file (matrix) | svg |
| 214 | A C T G G A T C C T G A A T C G A G T C T A G C C T G A C G T A T A C G A G T C C T A G C A G T T C A G T C G A T G A C G A T C | PAX5(Paired,Homeobox)/GM12878-PAX5-ChIP-Seq(GSE32465)/Homer | 1e-2 | -6.747e+00 | 0.0024 | 4130.0 | 4.11% | 3943.2 | 3.93% | motif file (matrix) | svg |
| 215 | T G C A T C G A T C G A A C G T C A T G C G T A A G T C T C A G A C G T G T A C C G T A A G C T | CREB5(bZIP)/LNCaP-CREB5.V5-ChIP-Seq(GSE137775)/Homer | 1e-2 | -6.453e+00 | 0.0032 | 5388.0 | 5.37% | 5182.8 | 5.16% | motif file (matrix) | svg |
| 216 | A C T G A G C T A G T C G T C A A G C T T C A G A T G C G A T C G C A T A T C G T C G A T A G C C G A T C A T G T A G C | Pax8(Paired,Homeobox)/Thyroid-Pax8-ChIP-Seq(GSE26938)/Homer | 1e-2 | -6.448e+00 | 0.0032 | 3878.0 | 3.86% | 3702.6 | 3.69% | motif file (matrix) | svg |
| 217 | C A T G G T A C C G T A A G T C C T A G A C G T A C T G G T A C A G T C A G C T | bHLHE40(bHLH)/HepG2-BHLHE40-ChIP-Seq(GSE31477)/Homer | 1e-2 | -6.398e+00 | 0.0034 | 2937.0 | 2.93% | 2784.3 | 2.77% | motif file (matrix) | svg |
| 218 | C G T A C T G A C T A G C G T A C G T A A G T C C G T A C A G T G C A T G T C A C G A T A C T G A C G T G C A T G A T C | PGR(NR)/EndoStromal-PGR-ChIP-Seq(GSE69539)/Homer | 1e-2 | -6.339e+00 | 0.0036 | 3055.0 | 3.04% | 2900.0 | 2.89% | motif file (matrix) | svg |
| 219 | C T A G A C T G T G C A G T C A A T G C C G T A A T C G A T G C A G T C C T A G | ZNF341(Zf)/EBV-ZNF341-ChIP-Seq(GSE113194)/Homer | 1e-2 | -6.263e+00 | 0.0038 | 8264.0 | 8.23% | 8018.4 | 7.98% | motif file (matrix) | svg |
| 220 | C A G T A C T G C G T A G T C A C G T A A G C T G A T C A G C T A C T G C G T A A G T C G T C A A G T C A G T C A T C G | Six4(Homeobox)/MCF7-SIX4-ChIP-Seq(Encode)/Homer | 1e-2 | -5.840e+00 | 0.0058 | 657.0 | 0.65% | 589.7 | 0.59% | motif file (matrix) | svg |
| 221 | G A C T A G C T G T A C G A C T C T G A A C T G G T C A C T G A A T G C T A C G G A C T A C G T A G T C G A C T C T G A | HRE(HSF)/Striatum-HSF1-ChIP-Seq(GSE38000)/Homer | 1e-2 | -5.705e+00 | 0.0066 | 3402.0 | 3.39% | 3250.9 | 3.24% | motif file (matrix) | svg |
| 222 | C T G A C T A G T C G A C G T A A T G C C G T A A T C G C G A T T A G C G C A T A T C G G C A T A G C T G A T C G A C T A G C T | ARE(NR)/LNCAP-AR-ChIP-Seq(GSE27824)/Homer | 1e-2 | -5.703e+00 | 0.0066 | 2832.0 | 2.82% | 2693.0 | 2.68% | motif file (matrix) | svg |
| 223 | T A C G T C G A G A C T A C T G C T G A A G T C T C A G G A C T T G A C C T G A | Atf1(bZIP)/K562-ATF1-ChIP-Seq(GSE31477)/Homer | 1e-2 | -5.490e+00 | 0.0081 | 8228.0 | 8.20% | 8004.9 | 7.97% | motif file (matrix) | svg |
| 224 | C A G T G A C T A G T C G C T A C T G A G T C A C G T A A G C T G T C A C T G A C T G A C T G A A C T G A G C T G A T C | MYNN(Zf)/HEK293-MYNN.eGFP-ChIP-Seq(Encode)/Homer | 1e-2 | -5.459e+00 | 0.0084 | 4194.0 | 4.18% | 4031.7 | 4.01% | motif file (matrix) | svg |
| 225 | T G C A T A G C G A C T T G C A T G A C T G C A C G T A A G C T A G C T A G T C A G T C G T A C | GFY(?)/Promoter/Homer | 1e-2 | -5.377e+00 | 0.0090 | 631.0 | 0.63% | 568.5 | 0.57% | motif file (matrix) | svg |
| 226 | T A G C C G A T A C G T A G C T A G C T A G T C A T G C A G T C A C T G A T G C A T G C G C T A | E2F7(E2F)/Hela-E2F7-ChIP-Seq(GSE32673)/Homer | 1e-2 | -5.359e+00 | 0.0092 | 758.0 | 0.76% | 689.2 | 0.69% | motif file (matrix) | svg |
| 227 | A C T G C A G T A C G T C G T A C G T A A C G T A C T G C T G A | Nkx6.1(Homeobox)/Islet-Nkx6.1-ChIP-Seq(GSE40975)/Homer | 1e-2 | -5.355e+00 | 0.0092 | 43168.0 | 43.00% | 42782.6 | 42.59% | motif file (matrix) | svg |
| 228 | T C G A C A G T A C T G A G C T C G T A C G T A A C G T A C G T C T G A T A G C | Dlx3(Homeobox)/Kerainocytes-Dlx3-ChIP-Seq(GSE89884)/Homer | 1e-2 | -5.337e+00 | 0.0093 | 12113.0 | 12.07% | 11853.9 | 11.80% | motif file (matrix) | svg |
| 229 | T C A G T G A C G T A C C G T A A C G T T G A C A C G T T C A G A G C T G A C T | NeuroD1(bHLH)/Islet-NeuroD1-ChIP-Seq(GSE30298)/Homer | 1e-2 | -5.294e+00 | 0.0096 | 10486.0 | 10.44% | 10243.6 | 10.20% | motif file (matrix) | svg |
| 230 | A G T C A G T C C G A T A C G T A C G T A C T G A C G T A G C T A G T C A G T C | Sox4(HMG)/proB-Sox4-ChIP-Seq(GSE50066)/Homer | 1e-2 | -5.270e+00 | 0.0098 | 11931.0 | 11.88% | 11675.5 | 11.62% | motif file (matrix) | svg |
| 231 | C A G T T C A G A G C T G A C T A C G T A G T C G A T C G A C T C T G A A C T G G A T C C G T A C T G A A G T C G T A C | Rfx6(HTH)/Min6b1-Rfx6.HA-ChIP-Seq(GSE62844)/Homer | 1e-2 | -5.187e+00 | 0.0107 | 15121.0 | 15.06% | 14842.0 | 14.78% | motif file (matrix) | svg |
| 232 | A G C T C G A T T A C G A T C G A T C G C A G T A G T C A G T C A C T G T A G C | HINFP(Zf)/K562-HINFP.eGFP-ChIP-Seq(Encode)/Homer | 1e-2 | -5.003e+00 | 0.0127 | 2404.0 | 2.39% | 2287.5 | 2.28% | motif file (matrix) | svg |
| 233 | C T G A T C A G G C T A A G C T A G T C G A C T C T G A C T A G T G C A C T G A A G T C G T A C G A T C A C T G T C G A | ZBTB12(Zf)/HEK293-ZBTB12.GFP-ChIP-Seq(GSE58341)/Homer | 1e-2 | -4.848e+00 | 0.0148 | 5488.0 | 5.47% | 5319.0 | 5.30% | motif file (matrix) | svg |
| 234 | C T A G T A G C A G T C G T C A C T G A A C G T C G T A C G T A C G T A G C T A | Hoxd13(Homeobox)/ChickenMSG-Hoxd13.Flag-ChIP-Seq(GSE86088)/Homer | 1e-2 | -4.836e+00 | 0.0149 | 29434.0 | 29.32% | 29101.6 | 28.97% | motif file (matrix) | svg |
